# Abnormal Blood Glucose as a Prognostic Factor for Adverse Clinical Outcome in Children Admitted to the Paediatric Emergency Unit at Komfo Anokye Teaching Hospital, Kumasi, Ghana

**DOI:** 10.1155/2014/149070

**Published:** 2014-12-28

**Authors:** Emmanuel Ameyaw, Kwame Amponsah-Achiano, Peter Yamoah, Jean-Pierre Chanoine

**Affiliations:** ^1^Komfo Anokye Teaching Hospital, P.O. Box 1934, Kumasi, Ghana; ^2^Disease Control Unit, Ghana Health Service, Accra, Ghana; ^3^Endocrinology and Diabetes Unit, British Columbia Children's Hospital, Room K4-212, 4480 Oak Street, Vancouver, BC, Canada V6H 3V4

## Abstract

Dysglycaemia (hyper- or hypoglycaemia) in critically ill children has been associated with poor outcome. We compared the clinical outcomes in children admitted to Pediatric Emergency Unit (PEU) at Komfo Anokye Teaching Hospital (KATH) for acute medical conditions and presenting with euglycaemia or dysglycaemia. This is a prospective case matching cohort study. Eight hundred subjects aged between 3 and 144 months were screened out of whom 430 (215 with euglycaemia and 215 with dysglycaemia) were enrolled. The median age was 24 months (range: 3–144 months). In the dysglycaemia group, 28 (13%) subjects had hypoglycemia and 187 (87%) had hyperglycemia. Overall, there were 128 complications in 116 subjects. The number of subjects with complications was significantly higher in dysglycaemia group (*n* = 99, 46%) compared to euglycaemia group (*n* = 17, 8%) (*P* < 0.001). Forty subjects died out of whom 30 had dysglycaemia (*P* = 0.001). Subjects with dysglycaemia were 3 times (95% CI: 1.5–6.0) more likely to die and 4.8 times (95% CI: 3.1–7.5) more likely to develop complications (*P* = 0.001). Dysglycaemia is associated with increased morbidity and mortality in children with acute medical conditions and should lead to intensive management of the underlying condition.

## 1. Introduction

In children, the response to the stress of an acute illness can present as abnormal blood glucose [1–6]. Bhisitkul et al. [3] found that 3.8% of children and adolescents presenting to the emergency room in Norfolk (VA) had hyperglycaemia. Hyperglycaemia was not associated with a specific diagnosis category but reflected a greater severity of the underlying condition. In contrast, Elusiyan et al. [4] observed that 6.4% of pediatric subjects admitted to a hospital in Nigeria had hypoglycaemia. Similarly, hypoglycaemia was observed across a large range of conditions and was associated with increased mortality. Overall, hypoglycaemia as well as hyperglycaemia [1–5] and, in addition, increased glucose variability [7] have been found to be associated with increased morbidity and mortality rates in acutely ill children in a variety of settings.

There is presently no data on abnormal blood glucose concentrations as well as on their potential relationship with patient outcome, in children with acute illnesses admitted to the Pediatric Emergency Unit (PEU) at KATH. We hypothesized that both hypoglycaemia and hyperglycaemia (referred to as dysglycemia in this paper) would be observed in children presenting to PEU and would be associated with unfavourable outcome. Using a prospective, case match design, the objectives of this study were (1) to describe and compare the characteristics of subjects as a function of their baseline blood glucose concentration and (2) to assess dysglycaemia as a prognostic factor for adverse clinical outcome in children admitted to PEU at KATH, Kumasi, Ghana.

The long term goal of our project is to learn from the results of this study to help develop and implement appropriate policies and treatment protocols and, ultimately, to better manage children at risk of complications. This could decrease morbidity and mortality and, ultimately, increase treatment efficiency and decrease treatment costs.

## 2. Material and Methods

### 2.1. Study Population

The study was conducted on consecutive subjects presenting over a four-month period to PEU, between December, 2010, and March, 2011. All children admitted with acute illness were eligible to participate in this prospective study. Inclusion criteria were admission to PEU with acute illness and age between 3 months and 12 years. Exclusion criteria were known history of diabetes mellitus; infusion of dextrose containing fluids up to 2 hours prior to admission; and intake of steroids within 72 hours of admission.

### 2.2. Study Site

The study was conducted at the PEU of the Department of Child Health, KATH, Kumasi. KATH is a 1200-bed capacity tertiary hospital which serves the Kumasi Metropolis and the surrounding towns and villages and also serves as a referral center for the middle belt and the northern part of Ghana. It has a polyclinic department which takes care of the outpatient cases. The paediatric unit of the polyclinic accepts children from birth to 12 years of age. Children who are critically ill are admitted from the polyclinic to the wards through the PEU. Both the polyclinic and the wards run a 24-hour duty every day. The number of admissions to PEU is between 15 and 30 patients everyday (unpublished data). Patients admitted to PEU are monitored till they are stable and then they are transferred to the wards for further treatment till they are well enough to be discharged.

### 2.3. Study Procedure

Data were collected daily between 8 a.m. and 8 p.m. All subjects admitted to PEU with acute illnesses had their blood glucose checked with One Touch Ultra2 glucometer (Lifescan Company, Milpitas, CA, USA). The range of the glucose concentrations measured with One Touch Ultra2 glucometer is 0.6–33.3 mmol/L (10–600 mg/dL). Glucometers were calibrated at the KATH Biochemistry Laboratory for every twentieth patient. A difference of less than 10% between that measure by glucometer and that by the biochemistry laboratory was regarded as acceptable [8]. Hyperglycaemia and hypoglycaemia were defined as blood glucose greater than 8.3 mmol/L (150 mg/dL) and less than 2.5 mmol/L (45 mg/dL), respectively [1–4].

A subject with acute illness and abnormal blood glucose (dysglycaemia group) was matched to the next patient with normal blood glucose (euglycaemia group) but with the same diagnosis category (Table 3) and age group (age from 3 months to <2 years, 2 to <5 years, and 5 to 12 years) in order to better compare the characteristics of the conditions associated with dysglycemia. They were followed up till discharge or death. On discharge, their folders were collected and case record form was completed for final diagnosis and complications developed including death. Subjects in whom hyperglycemia did not resolve or worsened after initiating specific treatment of the primary condition were treated with regular insulin and monitored closely [9]. In contrast, those with hypoglycaemia were managed immediately according to our local protocol. Our hospital protocol calls for an initial bolus intravenous (iv), intraosseous (io), or rectal dextrose or intramuscular (im) glucagon followed by i.v. 10% dextrose at maintenance. Feeding is resumed as soon as possible. Necessary supportive management was given to each patient.

### 2.4. Data Collection and Analysis

Data were collected on CRF which was designed to capture demographic (age, sex) and clinical data and outcome. Data were entered onto a predesigned electronic CRF using Epi-Info version 3.5.1. and transferred to Stata version 10 (StataCorp, TX, USA), for analysis. Descriptive analysis of baseline characteristics was compared in both groups. Exposure-outcome relationship was explored using univariate, bivariate, stratified, and multivariate analysis as appropriate. Risks of adverse outcomes in both groups were calculated and compared using risk ratios with 95% confidence intervals. Student's *t*-test was used to assess differences in the means of continuous variables and significant levels were assessed using *P* value of <0.05.

### 2.5. Ethical Considerations

A written informed consent form (ICF) detailing the study purpose, benefit, and possible risks to participants and their caregivers was provided. Authorization to conduct the study was obtained from the Head of Department of Child Health and the Medical Director of KATH. Ethical clearance was obtained from the Committee on Human Research Publication and Ethics of the Kwame Nkrumah University of Science and Technology, Kumasi, Ghana. Written informed consent was received from the parents/caregivers of the patients. Assent was sought from patients older than 8 years.

## 3. Results

Over the course of 4 months, 215 subjects with dysglycaemia were recruited and matched for age group and diagnosis category with 215 euglycaemic subjects. In total, 800 subjects were screened (546 euglycemic, 34 hypoglycaemic, and 220 hyperglycaemic).

Tables 1 and 2 describe the demographic and clinical characteristics of the 430 subjects with euglycaemia and with dysglycaemia. The median age of the subjects was 24 months (range: 3–144 months). The proportion of males and females was similar (60% boys in the euglycemic group and 55% boys in the dysglycaemia group). In the dysglycaemia group, 28 (13%) out of 215 subjects had hypoglycemia and 187 (87%) had hyperglycemia. Table 3 describes the medical conditions observed in the 430 subjects enrolled in the study. In keeping with the case-matching design of the study, the age distribution and the diagnosis categories were identical in euglycaemic and dysglycaemic subjects.

Table 4 describes the complications of the subjects at admission. Overall, there were 128 complications in 116 subjects. The number of subjects with complications was significantly higher in the dysglycaemia group (*n* = 99; 46%) compared to the euglycaemia group (*n* = 17; 8%) (*P* < 0.001). In the dysglycemia group, the proportion of subjects with complications was similar for hypoglycemia (13/28 subjects = 46%) and hyperglycemia (86/187 = 46%).

Table 5 shows the outcome at discharge of the subjects. The majority of the subjects (*n* = 382, 88%) had no complications at discharge and 8 (2%) were discharged with one or more complications. Six of them were in the dysglycemia group (hyperglycemia) (*P* < 0.001). Those who were discharged with complications were followed up till resolution. At the end of the study, 40 subjects had died. Death was statistically more common in subjects with dysglycaemia (*n* = 30) at admission than in euglycaemic subjects (*n* = 10) (Chi square test with Yates correction, *P* < 0.003). Among the 30 subjects with dysglycemia who died, 20 presented with hyperglycemia (=11% of the subjects with hyperglycemia) and 10 with hypoglycemia (=36% of the subjects with hypoglycemia) (Chi square test with Yates correction, *P* = 0.001). The risk of dying or of developing complications for subjects with dysglycaemia was, respectively, 3 times (95% CI: 1.5–6.0) and 4.8 times higher (95% CI: 3.1–7.5) than for euglycaemic subjects (*P* < 0.001).

## 4. Discussion

The results of our study show that both hypoglycemia and hyperglycemia reflect increased severity of an acute medical condition in children presenting to the Emergency Room at KATH.

These figures are similar to findings in other studies. Elusiyan et al. [4] found out that presence of hypoglycaemia at admission was associated with death and dying within 24 hours of admission. Moreover, Osier et al. [5] in Kenya found out that mortality for children with abnormal blood glucose was 34.2% compared to 7.6% in euglycaemic children admitted to an emergency ward. Furthermore, Dungan et al. [9] demonstrated that mortality in hyperglycaemic children was about twice as much as that in euglycaemic children even though the correlation between blood sugar and mortality could not be established. Additionally, a study in Mozambique revealed that mortality in children with acute medical disease was 16.3% in children with hypoglycaemia compared to 3.2% in those who were normoglycaemic [10].

Accordingly, we found out that subjects with dysglycaemia were 3 times more likely to die and 4.8 times more likely to have complications than those with euglycemia. Solomon et al. [11] found a relative risk of 5.8 for mortality in children with acute medical conditions and hypoglycaemia in Mozambique. The relative risk of dying of hypoglycaemia in the Mozambique study was about twice that of this study. Whilst the sample sizes of the Mozambique study (603 children) and our study were similar, the different results may be explained by differences in the selection of the study participants. Overall, the value of dysglycemia as a prognostic factor for mortality and morbidity seems well established.

In our study, the commonest disease found to be associated with dysglycaemia was severe malaria. This is not surprising as malaria is a common infection in most parts of Africa, including Ghana. The other common disease conditions we encountered were diarrhea and vomiting, acute respiratory diseases, and septicaemia. Various diseases have been established in other studies to be associated with abnormal blood glucose including severe malaria [12–15], gastroenteritis [16], septicaemia [17, 18], pneumonia [19], and acute febrile seizures [2]. Elusiyan et al. [4] in Nigeria found that hypoglycaemia was associated with severe malaria, septicaemia, pneumonia, and protein energy malnutrition among children admitted to a paediatric emergency ward.

Looking more in depth at malaria, the most common condition identified in this study, the link between dysglycaemia and severity of the disease is not completely understood. In a mostly pediatric population, Eltahir et al. [20] observed hyperglycemia in severe cerebral malaria which was secondary to an increase in insulin resistance and, possibly, to a decrease in insulin production. On the other hand, hypoglycemia is also commonly reported in children with severe malaria, more so in Africa than in other continents [21]. The reason is unclear and may reflect differences in nutritional status, infection itself, genetic predisposition, or preadmission use of quinine. Indeed, quinine, which is commonly used for treatment of severe malaria in many parts of Africa, including Ghana, has been suggested to affect insulin secretion. Abdallah et al. [22] observed hypoglycemia in 12% of adult subjects receiving intravenous quinine for severe malaria. However, Kawo et al. [23] did not observe significant changes in blood glucose in subjects with falciparum malaria receiving intravenous administration of quinine.

Putting together, these data suggest that early recognition of dysglycaemia in subjects with acute illness should prompt the health professional to adopt a more aggressive management approach.

Several issues remain to be clarified. First, there is a need for a better definition of the severity of hypoglycemia with regard to the increased risk of morbidity and mortality [24]. Second, whether correction of dysglycemia in addition to aggressive management of the underlying condition is beneficial to the subject remains unclear. However, administration of glucose is recommended by the WHO [25] and advocated by some authors [11, 12, 26]. A recent review on the effect of acute hypoglycemia management in children suggests that the evidence remains insufficient and that longer term studies are needed [27]. Similarly, two recent reviews did not find that, although good glycemic control may improve outcome [28, 29], the role of hyperglycemia management in critically ill children was clearly demonstrated [30].

A strength of our study is the prospective, case matched cohort design. Previous studies that compared the health outcomes according to glycaemia at admission did include all patients without attempt to matching. As dysglycaemia is much less common than euglycaemia, this resulted in groups of very different sizes and etiologies [4, 5]. While the latter design allows for a description of the epidemiology of subjects with dysglycemia, our design is stronger when it comes to comparing the outcomes.

## 5. Conclusion

Abnormal blood glucose (dysglycaemia) is associated with increased morbidity and mortality in children with acute medical conditions on admission. Fatal outcome was more common in those who are hypoglycemic at admission. We suggest that all children with acute medical condition should be screened for dysglycaemia at admission and that abnormal blood glucose should promote aggressive management of the underlying condition. The role of hypo- or hyperglycemia management in the overall treatment plan remains unclear.

## Figures and Tables

**Table 1:** Demographic characteristics.

Age (months)	Euglycaemia *n* (%)	Dysglycaemia *n* (%)		
		All	Hypoglycemia	Hyperglycemia
3.0–23.9	101 (47)	101 (47)	17 (61)	84 (45)
24.0–59.9	58 (27)	58 (27)	5 (18)	53 (28)
60.0–144.0	56 (26)	56 (26)	6 (21)	50 (27)

All ages	215	215	28	187

Data are given as *n* (% of the patients with euglycemia or with dysglycemia (all, hypoglycemia and hyperglycemia)).

**Table 2:** Clinical features of children presenting with acute medical conditions at PEU (*n* = 430).

Symptom^*^	Frequency *n*	Percent (%)
Fever	377	88
Vomiting	289	67
Diarrhea	125	29
Poor feeding	227	53
Convulsion	110	26
Cough	148	35
Rhinorrhoea	77	18
Other symptoms^**^	230	54
Clinical signs		
Flaring	84	20
Intercostal recessions	57	13
Other clinical signs^***^	41	10

^*^Subjects can present with one or more symptoms or clinical signs.

^**^“Other symptoms” included chills, bodily pains, repeated convulsions, earache, ear discharge, painful throat, dysuria, headache, and abdominal pain.

^***^“Other clinical signs” included inflamed tonsils, pallor, abdominal tenderness, and inflamed eardrum.

**Table 3:** Categories of diagnosis in children presenting with acute medical conditions at PEU (n = 430).

Diagnosis	Frequency (*n*)	Percent (%)
Severe malaria	162	38
Severe malaria with acute disease	22	5
Assumed severe malaria	50	12
Septicaemia	40	9
Enteric fever	14	3
Meningitis	16	4
Diarrhea and vomiting	46	11
Acute respiratory disease	46	11
Sickle cell disease with acute disease	28	6
Others	6	1

Malaria with acute disease: malaria with one of the following: septicaemia, enteric fever, meningitis, or pneumonia.

Acute respiratory disease: acute asthma attack, pneumonia, or bronchiolitis.

Assumed severe malaria: patient with acute illness treated as severe malaria but not malaria parasites on blood film and negative blood, urine, and/or cerebrospinal fluid cultures. These patients improved on an antimalaria treatment.

Sickle cell disease (SCD) with acute disease: SCD with one of the following, severe malaria, severe anaemia, acute haemolysis, acute chest syndrome, septicaemia, and vasooclusive crisis.

Others: urinary tract infection, tonsillitis, and otitis media.

**Table 4:** Complications among the participants.

	All	Euglycaemia *n* (%)	Dysglycaemia *n* (%)		
			All	Hypoglycemia	Hyperglycemia
DIC	9 (2)	1 (0.5)	8 (4)^#^	3 (11)	5 (3)
ARF	17 (4)	2 (1)	15 (7)^#^	4 (14)	11 (6)
Shock	39 (9)	4 (2)	35 (16)^#^	6 (21)	29 (16)
Hepatitis	8 (2)	2 (1)	6 (2)	0 (0)	6 (3)
IVH	28 (7)	6 (3)	22 (10)^#^	0 (0)	22 (12)
Other complications^*^	27 (7)	2 (2)	25 (11)^#^	3 (11)	22 (12)

DIC: disseminated intravascular coagulation; ARF: acute respiratory failure; IVH: intraventricular hemorrhage.

^*^“Other complications” include cortical blindness, hemiparesis, haemoglobinuria, heart failure, hypoglycaemia, intestinal perforation, and repeated convulsions.

^#^ *P* < 0.05 compared to corresponding group with euglycemia.

Data are given as *n* (%). % reflects the percentage of complications in all subjects (*n* = 430), in subjects with euglycemia (*n* = 215) and in subjects with dysglycemia (all (*n* = 215), hypoglycemia (*n* = 28), or hyperglycemia (*n* = 187)). There were 128 complications in 116 subjects.

**Table 5:** Outcome at discharge.

Outcome	All *n* (%)	Euglycaemia *n* (%)	Dysglycaemia *n* (%)		
			All	Hypoglycemia	Hyperglycemia
Discharge without complications	382 (89)	203 (94)	177 (83)	18 (64)	159 (85)
Discharge with complications	8 (2)	2 (1)	6 (3)	0 (0)	6 (4)
Death	40 (9)	10 (5)	30 (14)	10 (36)	20 (11)

All	430 (100)	215 (100)	215 (100)	28 (100)	187 (100)
